# Inverse associations between Mediterranean diet constituents and the gut microbiota in metabolic-associated steatotic liver disease (MASLD): a case-control study

**DOI:** 10.1186/s12986-025-00939-8

**Published:** 2025-12-23

**Authors:** Georgina M. Williams, Emily C. Hoedt, Kerith Duncanson, Lay Gan, Emilia Prakoso, Nicholas J. Talley, Eleanor J. Beck

**Affiliations:** 1 College of Health, Medicine and Wellbeing, The University of Newcastle, Callaghan, NSW Australia; 2 NHMRC Centre for Research Excellence in Digestive Health, University of Newcastle, Newcastle, NSW Australia; 3 Hunter Medical Research Institute, New Lambton Heights, NSW Australia; 4 School of Health Sciences, University of New South Wales, Sydney, NSW Australia; 5 Department of Gastroenterology, John Hunter Hospital, New Lambton Heights, NSW Australia; 6 Faculty of Medicine and Health, Sydney Medical School, University of Sydney, Sydney, Australia; 7 Department of Gastroenterology and Liver, Liverpool Hospital, Sydney, NSW Australia; 8 AW Morrow Gastroenterology and Liver Centre, Royal Prince Alfred Hospital, Sydney, NSW Australia; 9 Hunter Medical Research Institute, Lot 1 Kookaburra Cct, New Lambton Heights NSW 2305, Georgina, Australia

**Keywords:** Microbiota, Microbiome, MAFLD, MASLD, NAFLD, Diet, Mediterranean diet

## Abstract

**Background:**

Dietary therapy, specifically for weight loss, is currently considered first-line therapy for metabolic-associated steatotic liver disease (MASLD). However, increasing recognition of the role of the gut-liver axis in MASLD highlights potential for microbiota-modulating dietary therapy to improve outcomes. This study aimed to explore dietary variables relevant to gut microbiota in MASLD.

**Methods:**

Twenty-five adults with MASLD and 25 healthy controls were recruited using a retrospective case-control design and characterised using 3-day dietary intake records, clinical markers, and shotgun metagenomic sequencing.

**Results:**

MASLD participants consumed less dietary fibre (p = < 0.01), very long chain omega-3 fatty acids (*p* = 0.02), nuts and seeds (*p* = 0.03), whole grains (*p* < 0.01) and vegetables (*p* = 0.04). Participants with MASLD had lower abundance of *Alistipes senegalensis* (*r*=-0.01, *p* = 0.04), *Coprococcus eutactus* (*r*=-0.07, *p* = 0.006), *Faecalibacterium* (*r*=-0.02, *p* < 0.001), and higher abundance of *Ruminococcus torques* (*r* = 0.04, *p* = 0.02), and less expression of functional pathways associated with ethanol production, methionine, folate and branched-chain amino acid metabolism. Bacterial species and functional pathways more abundant in MASLD were positively associated with intake of added sugars and saturated fat, and negatively associated with unsaturated fatty acid and dietary fibre intake.

**Conclusions:**

Microbiota characteristics differ between individuals with and without MASLD, and this is influenced by dietary intake. Future translation-focused research investigating dietary interventions and the gut-liver-axis in MASLD are warranted.

**Supplementary Information:**

The online version contains supplementary material available at 10.1186/s12986-025-00939-8.

## Background

More than 25% of the world’s adult population live with metabolic dysfunction-associated steatotic liver disease (MASLD) [1–2], characterised by hepatic steatosis and metabolic comorbidities [3]. MASLD was until recently known as non-alcoholic fatty liver disease (NAFLD), diagnosed based on the presence of hepatic steatosis and exclusion of excessive alcohol use and other diseases [4], however recent nomenclature change was adopted to reflect the synchronicity of metabolic syndrome and hepatic steatosis [5]. MASLD can progress to metabolic dysfunction-associated steatohepatitis (MASH) which increases risk of cirrhosis and hepatocellular carcinoma. Weight loss is a well-established component of MASLD management [6], with guidelines supporting a 3–10% body weight reduction for disease improvement, dependent on baseline body mass index (BMI) [7–8]. However, beyond an energy deficit for weight loss, evidence supporting dietary patterns or specific nutrients that exacerbate or ameliorate MASLD outcomes has been debated [9]. Most recently, dietary patterns aligned with Mediterranean principles, characterized by higher intakes of oily fish, fruits, vegetables, whole grains, nuts and legumes, and reduced intake of red meat, processed foods and added sugars, have been proposed to benefit hepatic outcomes [10–21].

Inflammation is a key factor in MASLD progression [3] and thus the anti-inflammatory nature of Mediterranean foods is proposed to elicit health benefits which may include reduced hepatic inflammation [22]. Furthermore, Mediterranean dietary patterns may indirectly benefit health outcomes by modulating the gastrointestinal microbiome. A Mediterranean-style diet is high in dietary fibre and phytonutrients, substrates for microbial fermentation, which promotes microbial diversity and alters metabolite production. These processes potentially minimize endotoxemia and subsequent inflammation, with beneficial effects on liver health [23]. Conversely, diets high in saturated fat and added sugars, including excess free fructose, are proposed to increase microbial dysbiosis, inflammatory metabolite production and impair liver metabolism, worsening hepatic inflammation and steatosis [24–27].

Emerging awareness of diet-microbiome interactions relevant to MASLD management coincides with increased understanding of the gut-liver axis. Facilitated by portal circulation, the gut-liver axis provides a bi-directional pathway for gut microbial metabolites to the liver, and liver-derived bile acids and immune factors to the small intestine, where they can modulate microbe composition and function [28]. Meta-analysis shows individuals with MASLD have higher abundance of *Escherichia*, *Prevotella* and *Streptococcus* and lower abundance of *Coprococcus*, *Faecalibacterium* and *Ruminococcus* compared to healthy controls [29]. These compositional differences likely contribute to differential metabolite production in individuals with MASLD, which can directly impact liver function [30].

Current literature addresses either the relationship between dietary intake or microbiome characteristics and MASLD outcomes, but not the potential relationship between diet, the microbiome and MASLD status. Specifically, most studies that interrogate the microbiome in MASLD do not collect detailed dietary intake data to examine specific diet-microbiome effects on MASLD. Current MASLD clinical guidelines lack recommendations regarding quantity and/or frequency of intake of specific foods and nutrients. Improved understanding of the intersecting roles of diet and the microbiome is needed to translate research into practical dietary recommendations in MASLD. The aim of this study was to investigate associations between diet, clinical markers and the microbiome in adults with MASLD compared to healthy controls, to inform future MASLD dietary interventions.

## Methods

### Study participants

A retrospective case-control study design was utilised to analyse and compare dietary intake, microbiome, and clinical markers of individuals with MASLD to healthy controls. All research was conducted in accordance with both the Declarations of Helsinki and Istanbul and ethical clearance was obtained from the South Western Sydney Ethics Committee (2020/ETH01641).

MASLD participants were recruited at gastroenterology clinics in Sydney and Newcastle, Australiabetween December 2019 and June 2021. Recruitment ceased due to COVID-19 restrictions, and this determined sample size. At the time of recruitment, the term NAFLD was accepted and Asia-Pacific guidelines for diagnosis based on abnormal liver enzyme tests, exclusion of other liver diseases and confirmed by ultrasound or biopsy were used to identify participants [4]. Eligible individuals were contacted by the study dietitian prior to their clinic appointment and informed of the study requirements. Interested patients were sent a written participant information and consent form which was collected at their next appointment, prior to collection of data for eligible and consenting participants. Inclusion criteria included English speaking adults (> 18 years of age) with diagnosed MASLD or MASH.

Control participants were age- and sex-matched one for one from the CRE Digestive Health Biobank (2020/ETH01635). Control participants provided one blood sample and routine liver function tests were conducted to confirm these were all normal. Inclusion criteria for control participants included English speaking adults (> 18 years of age) with no evidence of MASLD. In both MASLD and control participants, presence of gastrointestinal or autoimmune disease was an exclusion criteria. In both cohorts, no financial incentive was offered however participants were offered a free consult with the study dietitian.

### Sample collection

Participants were sent a study pack containing instructions for a weighed three-day food record and two Norgen stool nucleic acid collection and preservation tubes (Norgen Biotek Corp) and instructed to complete these prior to their appointment. Instructions were provided on how to accurately measure foods using scales. Anthropometrical measures were taken by clinic nurses using calibrated equipment. Demographic data including age, gender, medical history, smoking status, ethnicity, medications, supplements and physical activity were collected using electronic medical records and patient report when appropriate.

### Dietary data collection

Three-day food records were screened for completeness and missing detail was clarified between the study dietitian and participant. Food record data was entered into Foodworks 9 Professional (Xyris Software, Brisbane Australia) for analysis of nutrient intake using AUSNUT food databases. Food serve information was computed using Xyris software food serves based on the Australian Guide to Healthy Eating and food modelling approaches [31]. Whole grain and cereal fibre intake was calculated using the Australian whole grain database [32] and cereal fibre database [33]. Fructose intake was calculated by matching values available in the Australian Food Composition Database to foods in the AUSNUT 2011–2013 database (1406 values) and extrapolating to the remaining foods in the AUSNUT database using AUSNUT recipe files (i.e., using fructose value for each ingredient and determining proportion of this in mixed meal) and clinical judgement of study dietitians (e.g., fructose content of protein foods was assumed to be zero, fructose content of white bread assumed to be the same as wholemeal and grain bread varieties). Mean daily intake across all three days for each dietary variable was computed.

### MASLD assessment

Non-invasive tools (Fibrosis-4 Index for Liver Fibrosis; FIB-4) [34–36] and the MASLD Fibrosis Score (MFS) [37–39] were used to assess MASLD severity using data collected from medical records and reviewed by the study gastroenterologist.

### Statistical analysis of clinical data

Descriptive statistics were completed and reported as the mean and standard deviation for each group. All variables were tested for normality and homogeneity of variances using Shapiro Wilks and Levenes test respectively. Normally distributed data was compared between groups using an independent t-test or chi-squared test and Nonparametric Mann-Whitney U test was used when normality and equal variances could not be assumed using SPSS statistical package (version 29.0.1.0). Associations between dietary variables and MASLD clinical outcomes were assessed using Spearman’s correlations.

### Microbiome analysis

Stool samples were collected at home (Norgen Biotek Corp) and returned at clinic appointments and then stored at -80°C until processing. Stool was thawed prior to processing with 100-300 mg weighed out for total gDNA extraction. Samples were processed following the manufacturer’s instructions for the Faecal Microbiome DNA kit (Promega) and Maxwell RSC 48 System (Promega), with one modification. Samples resuspended with lysis buffer were homogenized for three-minute with silica bead (0.3 g of 0.1 mm and 0.1 g of 0.5 mm) using the Precellys 24 (Bertin) homogenizer at 5,000 rpm. Recovered DNA was sequenced on the NovaSeq6000 (Illumina) using NovaSeq6000, 2 × 150 bp paired-end chemistry according to the manufacturer’s protocol by Microba Life Sciences to a target depth of 3Gb per sample (approximately 7 M – 16 M paired-end reads).

Taxonomic assignment was completed with MetaPhlAn4, and functional assignment completed using HUMAnN3 MetaCyc pathways, both using default settings. Outputs from MetaPhlAn4 and HUMAnN3 were imported into RStudio (R v4.1.3). Phyloseq alpha diversity p-values were calculated for Shannons, Simpsons and Chao1 indices using a pairwise Wilcoxon test between groups adjusted using Benjamini Hochberg corrections. Beta diversity was determined using principal coordinates analysis (PCoA) using the Bray-Curtis index to measure variance between groups (AmpVis2). Statistical differences were compared using PERMANOVA (Adonis2) measures of dissimilarity. All values were considered statistically significant at *p* < 0.05. Multivariate associations with linear models (MaAsLin) were used to determine associations between dietary variables, clinical outcomes and relative abundances of phyla, family, genera, and species as well as Metacyc pathways. Confounders including age, gender, smoking status, prescription medications and BMI were controlled for within the model. A conservative q value cut off was set at < 0.02. The effect size (adjusted correlation coefficient) was calculated according to the formula, log(qval)*sign(coeff). Participants with missing dietary data were not included in microbiome analysis.

### Blood collection and analysis

At each appointment, two 40mL venous blood samples were drawn using ethylenediaminetetraacetic acid tubes and serum separating tubes. Samples were stored at -80°C until thawed for analysis. Interleukin 1β (IL-1β), interleukin-16 (IL-16) and tumour necrosis factor-alpha (TNF-α) were measured using Luminex bead based multiplex immunoassay system (Merck Milliplex high sensitivity T cell cytokine panel). Enzyme-linked immunosorbent assay was used to determine cytokeratin-18 (CK-18) levels (Peviva M30 Apoptosense). High sensitivity C-reactive protein (hs-CRP) was determined using Biobase BK400 hs-CRP assay.

## Results

### Participant characteristics

In total, 25 individuals with MASLD and 25 healthy controls were recruited (Table 1). Cardiometabolic risk factors were evident in 9 of 25 healthy control participants and 23 of 25 participants with MASLD. As such, 92% of participants in the disease group met criteria for updated nomenclature for MASLD. Age (*p* = 0.8) and gender (*p* = 0.5) did not differ between groups (Table 1). Height, weight, BMI, ethnicity and prescription medication use were statistically different between groups (all *p* < 0.05). Mean BMI in the MASLD group was consistent with an overweight weight classification (> 23 kg/m^2^ of Asian ethnicity or > 25 kg/m^2^ of non-Asian ethnicity) whilst mean BMI of the control group was within a healthy weight range (< 25 kg/m^2^) [40]. Ethnicity was associated with increased prescription medication use and higher TNF-a, so was controlled for in all analyses.

**Table 1:** Demographic and anthropometrical characteristics of participants with metabolic dysfunction associated steatotic liver disease (MASLD) (*n* = 25) and healthy controls (*n* = 25)

	MASLD (*n* = 25)	Controls (*n* = 25)	*P* value
Age in years (Mean ± SD)	52.0 ± 13.6	53.0 ± 12.8	0.8
Sex, n (%)			
Males	7 (28.0)	9 (36.0)	0.5
Females	18 (72.0)	16 (64.0)	
Ethnicity, n (%)			
Caucasian	9 (36)	26 (100)	**< 0.001**
Asian	7 (28)		
Middle Eastern	4 (16)		
Polynesian	1 (4)		
African	1 (4)		
South American	3 (12)		
Current smokers, n (%)	2 (8)	0 (0)	0.2
Regular prescription medications, n (%)	17(68)	2 (8)	**0.001**
Body Mass Index (kg/m^2^) of all participants^1^ (median (IQR))	27.2 (26.1–40.7)	24.6 (21.9–27.4)	**< 0.001**
Body Mass Index (kg/m^2^) of participants from Asian backgrounds^2^ (mean ± SD)	24.2 ± 4.1	N/A	
BMI > 25 kg/m^2^ (or 23 kg/m^2^ in participants from Asian backgrounds), n (%)	23 (92)	9 (36)	**< 0.001**
Dyslipidemia^3^, n (%)	6 (24)	1 (4)	**0.2**
Statin use, n (%)	6 (24)	1 (4)	**0.04**
Hypertension^4^, n (%)	9 (36)	0 (0)	**< 0.001**
Antihypertensive medication, n (%)	9 (36)	0 (0)	**< 0.001**
Insulin resistance^5^, n (%)	9 (36)	0 (0)	**< 0.001**
Antidiabetic medication, n (%)	8 (32)	0 (0)	**0.002**
Height (cm) (mean ± SD)	168.6 ± 11.6	166.9 ± 6.3	**0.02**
Weight (kg) (median (IQR))	84.0 (72.5-103.4)	75.0 (64.0-87.5)	**0.004**
Estimated Energy Requirement^6^ (mean ± SD)	8318.7 ± 1946.9	8255.6 ± 2023.2	0.9

^1, 2^BMI ≥ 25 kg/m2 [23 kg/m2 from Asian background] OR waist circumference > 94 cm (male) 80 cm (female) OR ethnicity adjusted for diagnosis of MASLD, ^3^Plasma triglycerides ≥ 1.70 mmol/L [150 mg/dL] OR lipid lowering treatment OR Plasma HDL-cholesterol ≤ 1.0 mmol/L [40 mg/dL] (M) and ≤ 1.3 mmol/L [50 mg/dL] (F) OR lipid lowering treatment for diagnosis of MASLD, ^4^Blood pressure ≥ 130/85 mmHg OR specific antihypertensive drug treatment for diagnosis of MASLD, ^5^Fasting serum glucose ≥ 5.6 mmol/L [100 mg/dL] OR 2-hour post-load glucose levels ≥ 7.8 mmol/L [≥ 140 mg/dL] OR HbA1c ≥ 5.7% [39 mmol/L] OR type 2 diabetes OR treatment for type 2 diabetes for diagnosis of MASLD, ^6^Based on Harris Benedict equation, physical activity level based on self-report, SD; standard deviation, IQR: interquartile range, *p* < 0.05 considered significant

In individuals with MASLD, FibroScan scores (*n* = 22) indicated mild hepatic fibrosis (7.35 ± 4.8). MFS and Fib-4 scores (*n* = 21) indicated the likelihood of liver scarring between fibrosis levels F0-F3 (nil- advanced fibrosis). Inflammatory markers hs-CRP (*p* = 0.01) and CK-18 (*p* = 0.03) were significantly higher in the MASLD group (Table 2).

**Table 2:** Blood fibrosis scores of individuals diagnosed with metabolic-associated fatty liver disease (MASLD) (*n* = 25) and serum inflammatory marker profiles of participants with MASLD and healthy controls

MASLD severity scores	MASLD	Controls	*P* value	Clinical indication
Fibroscan (*n* = 22) (mean ± SD)	7.35 ± 4.8	N/A	N/A	Normal to mild liver scarring
MASLD Fibrosis Score (*n* = 21) (mean ± SD)	-0.7 ± 1.75	N/A	N/A	Indeterminate score
Fibrosis-4 (mean ± SD) 36–64 years (validation population) (*n* = 17) > 65years (*n* = 4)	1.6 ± 1.6 1.86 ± 0.5	N/A	N/A	Fibrosis stage 2–3 Fibrosis stage 0–1
Hs-CRP (mg/L) (*n* = 49) (median (IQR))	4.7 (1.7–11.1)	1.8 (0.5–4.2)	**0.01**	N/A
CK18 (U/L) (*n* = 49) (median (IQR))	142.8 (83.8-308.9)	65.5 (52.8–83.0)	**0.03**	N/A
IL-1β (pg/mL) (*n* = 49) (median (IQR))	2.4 (1.3–3.4)	4.1 (2.2–6.3)	**< 0.001**	N/A
IL-6 (pg/mL) (*n* = 49) (median (IQR))	2.3 (1.1–6.3)	11.4 (1.1–31.1)	0.4	N/A
TNF-α (pg/mL) (*n* = 49) (median (IQR))	15.1 (11.8–19.6)	16.1 (13.4–21.2)	0.4	N/A

Reference ranges: ***FibroScan*** < 7.0 kPa No liver scarring or mild liver scarring, > 14 kPa likely cirrhosis, ***MASLD Fibrosis Score*** <-1.455 mild to no fibrosis, -1.455-0.675 indeterminate score (between mild-severe fibrosis), > 0.675 severe fibrosis to cirrhosis, ***Fibrosis-4*** < 1.3 advanced fibrosis excluded, < 1.3–2.67 approximate fibrosis stage 2–3, further investigation required, > 2.67 advanced fibrosis (METAVIR stage F3-F4) likely, ***ALT*** 7–55 IU/L, *IL-1β; Interleukin 1β*,* TNF-α; Tumour Necrosis Factor-a*,* CK-18; cytokeratin-18*,* hs-CRP; High sensitivity C-reactive protein (hs-CRP)*,* IL-6; interleukin-6*,* IQR: interquartile range*, p value significant at < 0.05

**Table 3:** Macronutrient and micronutrient intake of participants with metabolic associated steatotic liver disease and healthy controls measured using 3-day weighed food record

Dietary component	MASLD *n* = 25 (mean ± SD/median (range))	Control *n* = 23 (mean ± SD/median (range))	*P* value
Energy (kJ)	8598.7 ± 2892.9	9484.2 ± 2701.4	0.28
Percent EER (%)	110	107	0.65
Protein (g)	93.6 ± 28.4	98.2 ± 24.0	0.56
kJ from protein (%)	19.0 ± 4.1	18.1 ± 3	0.36
Total carbohydrate (g)	190.1 (165.1-256.3)	198.3 (163.7-254.7)	0.86
kJ from carbohydrate (%)	41.5 ± 8.6	36.5 ± 7.4	**0.03**
Sugars (g)	86.8 (55.7-106.2)	92.2 (63.2-106.2)	0.83
Added Sugars (g)	28.1 (17.8–57.6)	30.6 (12.6–38.4)	0.48
kJ from added sugars (%)	5.74 (3.34–11.9)	4.28 (1.81–7.71)	0.12
Free Sugars (g)	37.3 (21.7–61.2)	31.7 (15.4–48.6)	0.24
Dietary fibre (g)	19.5 (13.5–25.8)	27.6 (22.3–35.2)	**< 0.01**
kJ from fibre (%)	2.01 ± 0.8 (1.4–2.6)	2.5 ± 0.8	**0.03**
Alcohol (g)	0.00 (0.00–0.00)	0.00 (0.00-12.7)	**< 0.01**
kJ from alcohol (%)	0.00 (0.00–0.00)	0.00 (0.00-3.1)	**< 0.01**
Caffeine (mg)	114.2 (56.7-206.7)	163.4 (142.5–258.0)	**0.03**
Fructose (g)	17.2 (10.1–24.4)	15.0 (10.0-22.2)	0.63
Total fat (g)	69.7 (58.4–95.8)	105.3 (70.9-129.7)	**0.03**
kJ from fat (%)	34.0 (32.2–39.6)	38.8 (34.9–43.8)	**0.04**
Saturated fat (g)	23.7 (20.5–34.1)	30.6 (14.0-38.1)	0.60
Trans Fatty Acids (g)	1.10 (0.68–1.64)	1.7 (0.98–2.31)	0.09
Polyunsaturated fat (g)	11.3 (7.5–16.2)	15.1 (10.6–21.2)	0.07
Monounsaturated fat (g)	27.8 (22.7–36.3)	37.2 (24.5–54.7)	0.13
Very long chain omega-3 fatty acids (g)	0.15 (0.12–0.26)	0.26 (0.17–1.14)	**0.02**
F18D2CN6 linoleic acid (g)	10.3 (6.34–14.8)	12.7 (8.59–18.4)	0.11
F18D3N3 alpha-linolenic acid (g)	1.1 (0.67–1.42)	1.6 (1.30–1.92)	**< 0.01**
F20D5N3 eicosapentaenoic acid (g)	0.04 (0.02–0.94)	0.08 (0.04–0.41)	**0.03**
F22D5N3 docosapentaenoic acid (g)	0.07 (0.04–0.08)	0.13 (0.05–0.19)	**0.03**
F22D6N3 docosahexaenoic acid (g)	0.06 (0.03–0.13)	0.15 (0.04–0.42)	0.05

Estimated energy requirement (EER); kilojoules (kJ)

**Table 4:** Food group intake of participants with metabolic associated steatotic liver disease and healthy controls measured using 3-day weighed food record

Food serves^1^	MASLD *n* = 25 (mean ± SD/median (range))	Control *n* = 23 (mean ± SD/median (range))	*P* value
Protein food serves	2.9 ± 1.3	3.2 ± 1.5	0.48
Red meats	0.89 (0.49–1.56)	0.41 (0.00-1.37)	**0.04**
Poultry	0.35 (0.07–0.71)	0.11 (0.00-0.79)	0.17
Eggs	0.15 (0.03–0.33)	0.14 (0.01–0.33)	0.84
Processed meats	0.08 (0.00- 0.36)	0.06 (0.00-0.34)	0.92
Seafood high in long chain omega-3 fatty acids	0.00 (0.00-0.05)	0.01 (0.00-0.44)	0.06
Seafood low in long chain omega-3 fatty acids	0.00 (0.00-0.31)	0.00 (0.00-0.97)	0.39
Nuts and Seeds	0.10 (0.00-0.47)	0.37 (0.07–1.09)	**0.03**
Legumes	0.00 (0.00–0.00)	0.06 (0.00-0.45)	**0.02**
Soy products	0.00 (0.00–0.00)	0.00 (0.00–0.00)	0.90
Dairy serves	1.02 (0.35–1.66)	1.90 (1.13–2.44)	**< 0.01**
Milk	1.1 ± 2.4	1.1 ± 0.6	0.97
Cheese	0.18 (0.00-0.36)	0.40 (0.09–0.97)	**0.01**
Yoghurt	0.00 (0.00-0.26)	0.07 (0.00-0.30)	0.29
Grain food serves	5.7 ± 2.9	6.1 ± 2.0	0.60
Refined grains	4.8 ± 3.2	4.3 ± 2.7	0.56
Whole grains	0.9 ± 0.9	1.8 ± 1.4	**< 0.01**
Whole grain (dry) (g)^2^	23.6 (0.00-47.4)	46.9 (14.5–83.8)	**0.03**
Cereal Fibre (g)^2^	6.1 ± 2.8	8.8 ± 4.2	**0.01**
Added Sugars	10.3 ± 9.6	7.01 ± 5.09	0.36
Alcoholic Drinks	0.00 (0.00–0.00)	0.00 (0.00-1.27)	< 0.01
Fruit serves	1.05 (0.61–2.77)	1.21 (0.77–2.15)	0.72
Citrus melon berries	0.12 (0.00-0.44)	0.26 (0.02–0.45)	0.39
Fruit juice	0.00 (0.00-0.40)	0.00 (0.00–0.00)	0.44
Fruit juice (%)	0.00 (0.00–37.0)	0.00 (0.00–0.00)	0.21
Vegetable serves	3.03 ± 1.8	4.4 ± 2.6	**0.04**
Dark green vegetables	0.10 (0.01–0.25)	0.32 (0.02–0.70)	**0.05**
Red or orange vegetables	0.37 (0.15–0.67)	0.76 (0.39–1.31)	**0.02**
Tomatoes	0.16 (0.00-0.32)	0.22 (0.01–0.38)	0.37
Other red or orange vegetables	0.21 (0.03–0.26)	0.33 (0.23–1.02)	**< 0.01**
Starchy vegetables	0.74 (0.13–1.62)	0.85 (0.37–1.40)	0.92
Potatoes	0.62 (0.00-1.53)	0.59 (0.00-1.35)	0.83
Oil equivalents^3^	5.79 (3.63–8.81)	8.83 (5.72–17.2)	**0.01**
Solid fat equivalents^4^	11.0 ± 7.9	10.9 ± 5.3	0.97

^1^food serves as per AUSNUT 2011–2013 food composition database, ^2^ food serves as per whole grain and cereal fibre databases by Galea et al. [32] and Barrett et al. [33], ^3^oil equivalents: Fats naturally occurring in nuts, seeds, avocado, seafoods, and non-hydrogenated vegetable oils; excludes palm oil and coconut oil, ^4^solid fat equivalents: Fats naturally occurring in meat, poultry, eggs, dairy, fully or partially hydrogenated oils, shortening, palm oil and coconut oil

**Table 5:** Comparison of guidelines for dietary management of liver disease and study findings and implications for practice and future research

Area of practice	Clinical guidelines (AASLD)	Clinical guidelines (ESPEN)	Clinical guidelines (APASL)	Relevant outcomes from MASLD cohort in the current study	Implications for practice relevant to the microbiota in MASLD
Weight loss	Patients with NAFLD who are overweight or obese should be prescribed a diet that leads to a caloric deficit	In overweight/obese NAFL/NASH patients a 7–10% weight loss aimed for to improve steatosis and liver biochemistry; a weight loss of > 10% aimed for to improve fibrosis. To achieve weight loss, a hypocaloric diet followed according to current obesity guidelines irrespective of the macronutrient composition.	Overweight or obese and nonobese MAFLD can benefit from weight loss. In the former, a 7–10% weight loss is the target of most lifestyle interventions and results in improvement of liver enzymes and histology.	Energy intake (including % EER) did not differ between groups and was not associated with inflammatory markers or microbiota composition. Energy deficit was not evident, indicating dietetic intervention is warranted.	**Practice**: Dietetic input is likely necessary to ensure energy deficit in individuals with MASLD. Emphasis on diet quality within energy deficit is important to encourage foods which may beneficially modulate the microbiota.
Macronutrient composition	Diets with limited carbohydrates and saturated fat and enriched with high fibre and unsaturated fats (e.g., Mediterranean diet) encouraged due to their additional cardiovascular benefits.	A Mediterranean diet (MedD) advised to improve steatosis and insulin sensitivity. Until further data regarding their efficacy are available, omega-3-fatty acids cannot be recommended to treat NAFL/NASH.	Dietary recommendations consider energy restriction and exclusion of MAFLD-mediating components (processed food, food and beverages high in added fructose). A Mediterranean type of diet is advisable.	The predominance of associations between bacterial species and proportion of macronutrient intake may be indicative of relative substrate availability to the microbiota and subsequent fermentation. **Dietary carbohydrates**: The proportion of total kilojoule intake from carbohydrate was significantly higher in the MASLD group. Bacterial species were more consistently associated with kJ from carbohydrate, added and free sugars, than total carbohydrate. Species more abundant with higher intake of sugars were consistently positively associated with MASLD status and negatively associated with dietary fibre. 67% of the 340 associations between functional pathways and measures of carbohydrate intake related to added sugars. Whole food carbohydrate sources associated with bacterial species and functional pathways that were lower in MASLD group. **Dietary fats**: Intake of omega-3 fatty acids was significantly less in MASLD group. PUFA and omega-3 fatty acids consistently associated with microbe species and functional pathways that were less abundant in the MASLD cohort. Saturated fat intake did not differ between groups but was consistently associated with microbe composition, MASLD disease markers, inflammatory markers and functional pathways that were higher in MASLD group. **Dietary Fibre**: Significantly lower dietary fibre intake in MASLD group was consistently associated with less abundant microbe species and functional pathways. **Mediterranean Diet**: Mediterranean diet foods and nutrients were differentially consumed between groups. MASLD participants consumed less very long chain omega-3 fatty acids, dietary fibre, nuts and seeds, cheese, whole grains, dark green vegetables, red or orange vegetables and oils naturally derived from plant and seafood sources (excluding palm and coconut oil). These foods and nutrients were consistently negatively associated with MASLD-specific microbe composition, inflammatory markers and functional pathways. **Added Fructose**: Fructose intake did not significantly differ between groups fructose intake was associated with functional pathways enriched in the MASLD group and associated with Fib-4 and MFS.	**Practice**: Distribution of dietary carbohydrates and fat may be more relevant than total for microbiota-mediated outcomes. Balanced intake of macronutrients aligned with published acceptable macronutrient distribution ranges are suggested. **Dietary carbohydrates**: Associations between complex and refined carbohydrates and both microbiota characteristics and MAFLD status indicate carbohydrate type and quality affect microbiota-disease interactions. Focus on quality of carbohydrate foods in MASLD management, with low proportion of added and free sugars, and prioritise sources of carbohydrate associated with a Mediterranean diet pattern (legumes, vegetables, whole grains, fruit) encouraged. **Dietary Fats**: Type of dietary fat appears to modulate the gut microbiota, and correlate with disease status. Primarily source unsaturated fats in nuts and seeds, seafood high in omega-3 and plant-based oils. **Dietary Fibre**: Encourage whole food sources of dietary fibre in MASLD as this may enhance beneficial microbiota pathways, such as butyrate production. **Mediterranean Diet**: Mediterranean diet components that beneficially modulate the gut microbiota in MASLD are a key dietary management strategy. **Added Fructose**: Encourage individuals with MASLD to consume whole food forms of fructose such as fruit, in order to attain protective effects of the whole food matrix and associated nutrients such as dietary fibre.
Coffee	Coffee consumption (caffeinated or not) of at least 3 cups daily is associated with less advanced liver disease.			Caffeine intake in MASLD group equivalent to 2 serves of coffee each day and did not differ between groups. Caffeine intake was positively associated with *Bifidobacterium pseudocatenulatum*, which was also positively associated with MASLD status and hs-CRP and negatively associated with IL-1b.	The beneficial effect of coffee, from caffeine or phytochemicals, indicate that regular moderate coffee intake is desirable.
Alcohol	Patients with clinically significant hepatic fibrosis (≥ F2) should abstain from alcohol use completely	NAFL/NASH patients shall be encouraged to abstain from alcohol in order reduce risk for comorbidity and to improve liver biochemistry and histology	Patients with MAFLD should be advised to avoid alcohol and if that is not possible, to consume the lowest amount possible.	Alcohol intake was significantly higher in the control group, likely due to advice in the MASLD cohort to cease alcohol consumption. However, functional pathways associated with ethanol production were higher in MASLD group and associated with sugar intake and inflammatory markers. Alcohol intake was associated with 11 bacterial species, each of which were less abundant in the MASLD cohort, aligning with alcohol intake data. Alcohol intake was not correlated with any functional pathways.	Alcohol intake modulates the gut microbiota. Recommend that individuals with MASLD limit alcohol intake, and limit intake of dietary components that may enhance endogenous alcohol production such as monosaccharides fructose and glucose.

### Dietary intake

Twenty-five MASLD participants and 23 controls completed 3-day food records. Energy and total macronutrient intake were not significantly different between groups, however, the proportion of kilojoules from carbohydrates was significantly higher in the MASLD group (*p* = 0.03). Although not significant, the MASLD group consumed more carbohydrates from free sugars including fructose. Participants in the MASLD group consumed significantly less very long chain omega-3 fatty acids (VLCN3), alcohol and dietary fibre (all *p* < 0.05) (Table 3). Healthy controls consumed more serves of legumes, nuts and seeds, cheese, whole grains, alcoholic drinks, dark green vegetables, red or orange vegetables and oil equivalents (i.e., naturally occurring fats found in seafood and plant-based foods excluding palm and coconut oil) than MASLD participants (all *p* < 0.05) (Table 4). Red meat was the only food serve consumed significantly more in the MASLD group (*p* = 0.04). Alcohol intake was significantly higher in the control group (p = < 0.01), likely due to advice in the MASLD cohort to cease alcohol consumption and ethnicity differences. Mean alcohol intake in the control group was less than one standard drink per day (5 g/day) (Table 4).

Significant associations were evident between dietary nutrients, clinical and inflammatory markers (Supplementary Table 1). These included higher hs-CRP, IL-16, TNF-a and CK-18 with lower intake of polyunsaturated fat (PUFA), caffeine, VLCN3, dietary fibre, eicosapentaenoic acid (EPA), linoleic acid, docosahexaenoic acid (DHA), docosapentaenoic acid (DPA), legumes, nuts and seeds, wholegrain, fruit and vegetable serves and oil equivalents (all *p* < 0.05). Proportion of fat intake from saturated fats was positively associated with hs-CRP (*r* = 0.32, *p* = 0.03), Il-6 (*r* = 0.33, *p* = 0.03) with proportion of fat from polyunsaturated fats and VLNC3 negatively associated with hs-CRP (*r*=-0.39, p = < 0.01, *r*= -35, *p* = 0.02 respectively). Both Fib-4 and MFS were positively associated with fructose intake (*r* = 0.56, p = < 0.01 and *r* = 0.48, *p* = 0.03 respectively) (Supplementary Table 1).

### Microbiome taxonomic and functional MASLD signature

**Fig. 1:**
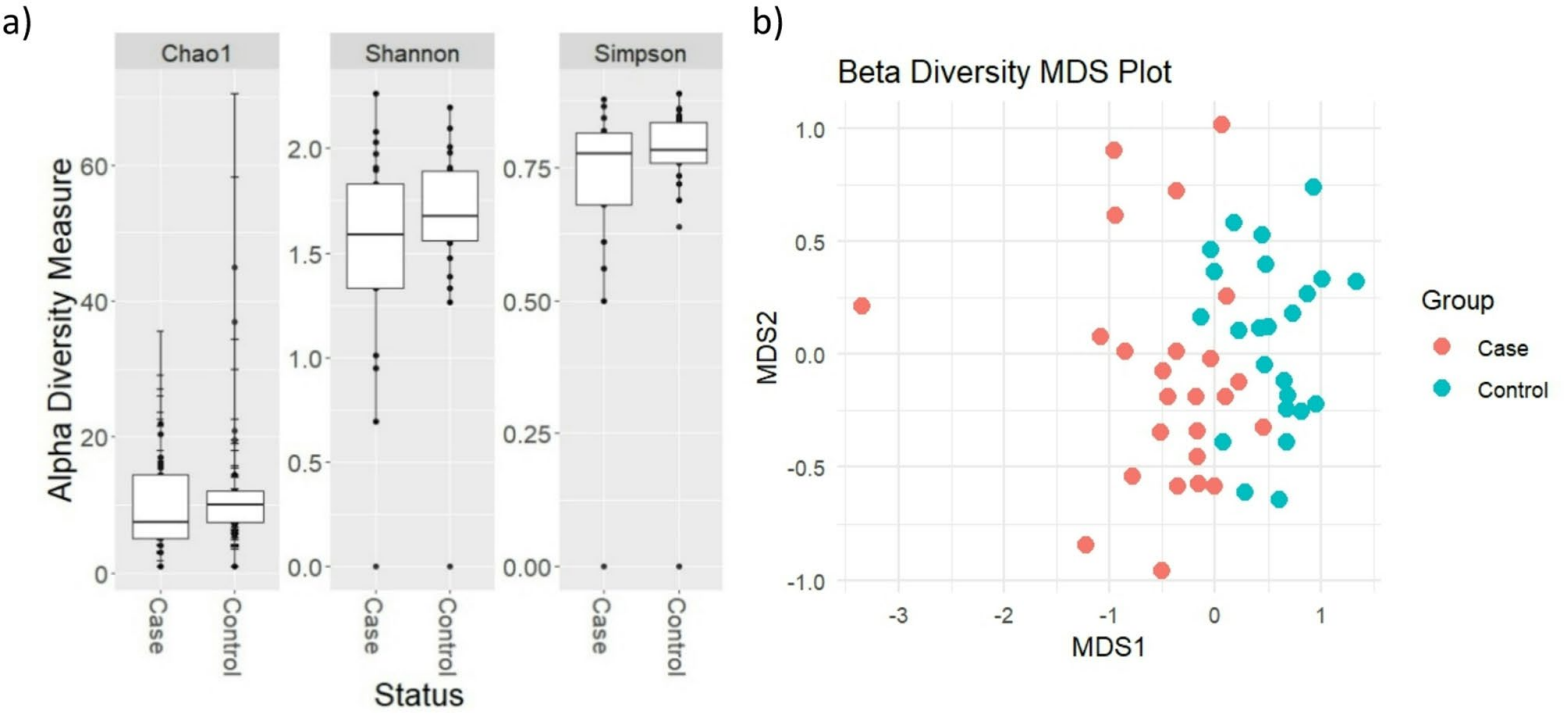
(**a**) Alpha diversity differences and (**b**) beta diversity PCoA plot based on Bray-Curtis index with PERMANOVA statistical analysis indicating dissimilarity between metabolic dysfunction associated steatotic liver disease (red) and control groups (blue)

**Fig. 2:**
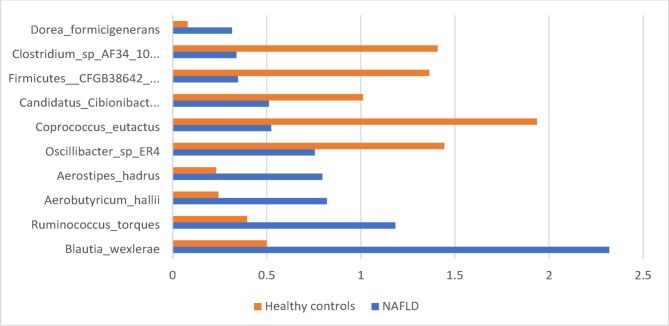
Bar chart depicting relative abundance (%) of the ten most abundant bacterial species identified in fecal samples obtained from 25 adults with non-alcoholic fatty liver disease (NAFLD) and 25 healthy adults (controls)

No significant difference in alpha diversity indices were observed between groups (Chao1 *p* = 0.11, Simpson *p* = 0.18, Shannon *p* = 0.22; Fig. 1a). However, beta diversity Bray-Curtis PCoA plot was significantly different between groups (PERMANOVA *p* = 0.001, R^2^ = 15%; Fig. 1b). Additionally, we identified 156 significant associations across different taxonomic levels relative abundance and MASLD status (Fig. 2, Supplementary Table 2), this included 38 statistically significant differences microbial species. No associations were evident between microbiota composition and participant factors: age, gender, BMI, prescription medication use or ethnicity.

Expression of 116 microbial functional pathways differed between MASLD cases and controls. 648 associations were evident between functional pathways and dietary variables, with 340 of these associated with measures of carbohydrate intake, 169 associated with dietary fat intake and 33 associated with dietary fibre intake (Supplementary Table 3). Functional pathways that were differentially expressed in individuals with and without MASLD were searched for pathways referenced in existing literature to be of relevance. Increased expression of pathways associated with ethanol production, methionine metabolism, folate metabolism and BCAA metabolism were evident in MASLD cohorts (Supplementary Table 3).

Microbes identified to differ between control and MASLD groups were investigated for relationships with functional pathways using HUMAnN data (Supplementary Tables 2 and 3). Twelve species that were significantly associated with MASLD status were identified to contribute to the expression of pathways enriched in the MASLD group, including those associated with acetate, lactate, formate and ethanol production and heme, butanediol, molybdopterin and tetrapyrrole synthesis. These pathways were also associated with increased intake of protein, added sugar, free sugar, total carbohydrates and negatively associated with dietary fibre, EPA, PUFA, dietary fibre, unsaturated oils, wholegrains, nuts and seed intakes. Inversely, pathways associated with bacterial species that were negatively associated with MASLD status included those associated with butyrate production, isoprenoid and folate metabolism, amino acid and ribonucleotide synthesis.

### Microbiome and dietary associations

**Fig. 3:**
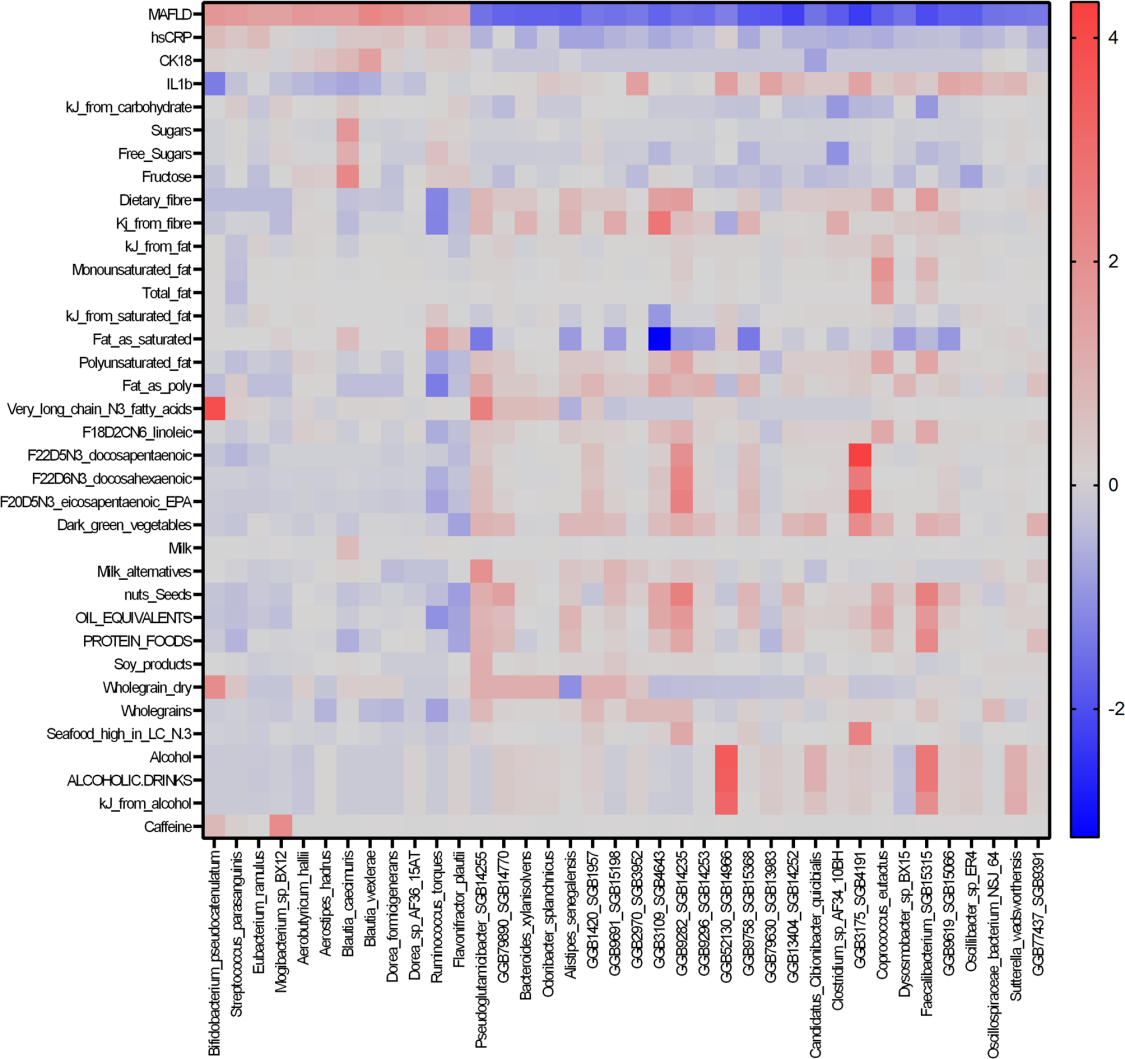
Associations between bacterial species, metabolic dysfunction associated steatotic liver disease (MASLD) status and dietary intake. IL-1β; Interleukin 1β, TNF-α; Tumour Necrosis Factor-a, CK-18; cytokeratin-18, hs-CRP; High sensitivity C-reactive protein (hs-CRP), IL-6; interleukin-6, kJ; kilojoules, N3; omega-3, oil equivalents: Fats naturally occurring in nuts, seeds, avocado, seafoods, and non-hydrogenated vegetable oils; excludes palm oil and coconut oil

One hundred and fifty-three associations were evident between dietary components and microbial species. Of note were associations identified with dietary fat intake (54 associations), dietary fibre (15 associations), alcohol (11 associations), measures of sugar intake (5 associations) and caffeine (2 associations) (Fig. 3). Species that were associated with higher unsaturated fat intake were less abundant in individuals with MASLD. These species included *Pseudoglutamicibacter* SGB14255, *Coprococcus eutactus*,* Faecalibacterium* SGB15315, *Dysosmobacter* sp. BX15 and unclassified species of Enterococcaceae, Firmicutes, and Clostridiaceae. *Alistipes senegalensis* and unclassified species of Bacteroidetes also exhibited this association pattern with dietary fats and MASLD status, and both were also negatively associated with hs-CRP. Inversely, *Ruminococcus torques* was negatively associated with PUFA and EPA intake, and positively associated with saturated fat intake and MASLD status. Additionally, *Pseudoglutamicibacter* SGB14255, *Alistipes senegalensis*,* Coprococcus eutactus*,* Faecalibacterium* SGB15315 and unclassified species of Enterococcaceae and Firmicutes were positively associated with dietary fibre intake, while *Ruminococcus torques* was negatively associated with dietary fibre intake. Associations between sugar intake and bacterial species were apparent only for free sugar intake, and not added sugar. Notably, more associations were evident for markers of the proportion of macronutrient intake (e.g., kilojoules from saturated fat or percentage of total fat as PUFA than measures of total macronutrient intake e.g., total fat) (Fig. 3).

Species relative abundance was also associated with whole food intake including whole grains (15 associations), nuts and seeds (11 associations), dark green vegetables (12 associations), protein foods (8 associations), seafood high in VLNC3 (2 associations) and oil equivalents (11 associations) (Fig. 3). Associations between food serves and bacterial species reflected nutrient associations. Again, *Pseudoglutamicibacter* SGB14255, *Alistipes senegalensis*,* Coprococcus eutactus*,* Flavonifractor plautii*,* Faecalibacterium* SGB15315 and unclassified species of Clostridiaceae and Firmicutes were positively associated with intake of unsaturated fat and dietary fibre intake and positively associated with intake of foods rich in these nutrients including dark green leafy vegetables, whole grains, plant-based milks, soy products, protein serves, nuts and seeds and oil equivalents. *Ruminococcus torques* was negatively associated with whole grain and oil equivalents intake. Unclassified species of Clostridia, and *Oscillospiraceae bacterium* NSJ 64 were positively associated with multiple food serves including dark green leafy vegetables, protein serves, nuts and seeds and oil equivalents and not associated with any nutrients.

Finally, investigation of microbial functional pathways that were identified as increased in relative abundance in participants with MASLD were negatively associated with dietary fibre and fat intake and positively associated with total energy, measures of sugar (Supplementary Table 3). Whole food intake data showed associations between functional pathways and serves of nuts and seeds, organ meats and tomatoes (Supplementary Table 3).

### Microbiome associations with host markers of disease

Species abundance was associated with hs-CRP, IL-1b and CK-18, and not with MASLD scores (Fig. 3). Only one species (*Bifidobacterium pseudocatenulatum*) was associated with more than one inflammatory marker (positively associated with hs-CRP (*r* = 0.73, *p* = 0.006) and negatively associated with IL-1β (*r*= -1.3, *p* = 0.007). In addition, CK-18 was positively associated with 8 microbial functional pathways and IL-1β was associated with 37 pathways. Fib-4 and MFS scores were not associated with functional pathway expression (Supplementary Table 3).

## Discussion

The current study supports evidence that microbiota composition and function are different in individuals with and without MASLD and contributes novel analyses showing that dietary intake influences these associations. This indicates that dietary intervention aimed at modulating the microbiota may benefit MASLD outcomes. Notably, dietary constituents that were consistently associated with bacterial species and functional pathways less abundant in the MASLD cohort, included components generally considered healthier dietary choices and specifically, elements of a Mediterranean dietary pattern. This supports recent evidence that Mediterranean diet principles are likely beneficial in MASLD [10–41], and suggests these benefits are attributable, at least in part, to their interactions with gut microbes and their subsequent functions.

Microbiota composition findings in our study support outcomes of meta-analysis [29], observational [42–44] and animal studies [45] assessing microbial composition in MASLD. Furthermore, studies assessing the influence of dietary components key to a Mediterranean dietary pattern, such as dietary fibre, on microbiota composition have indicated similar patterns of species abundance [43, 46, 47]. Whilst differences in species composition between disease states and healthy individuals suggests the microbiota influences disease mechanisms, metagenomic and metabolomic data is necessary to understand function. Our study supported hypotheses of altered ethanol, short chain fatty acids (SCFA), amino acid and folate metabolism in MASLD [28, 48−52], with increased expression of pathways associated with ethanol and butyrate production and methionine, folate and BCAA metabolism in the MASLD group. Individuals with MASLD have increased ethanol detectable in the portal vein [53] and increased abundance of ethanol producing bacteria such as *Klebsiella pneumoniae* [54]. In vitro research has demonstrated that both fructose and glucose metabolism can promote microbial ethanol production, but fermentation of polysaccharides and disaccharides containing fructose and glucose did not produce substantial amounts of ethanol [55]. Furthermore, it has been suggested that increased intake of monosaccharides may promote rapid fermentative pathways that produce alcohols such as ethanol [56–57]. This is of interest as the current study found that only free sugar intake was associated with bacterial species differences between groups. These findings and the current study suggest that replacing free sugars with complex carbohydrates is likely to be useful for beneficial microbiota modulation.

Amino acid metabolism is of interest in MASLD with epidemiological and animal studies inconclusive as to whether BCAA are detrimental or beneficial to hepatic outcomes [58]. Our study found that BCAA synthesis pathways were increased in individuals with MASLD, and diet influenced expression, with BCAA synthesis positively associated with added sugar intake and negatively associated with intake of PUFA. These findings are consistent with RCT’s which have found dietary interventions alter BCAA metabolism, one of which reported BCAA synthesis increased proportionally to intrahepatic fat in 196 individuals with MASLD, yet expression of this pathway was mitigated by supplementation with dietary fibre [59]. In another RCT, provision of a Mediterranean diet, decreased BCAA synthesis and liver fat compared to a control diet [60–61] suggesting interactions between dietary intake and BCAA metabolism may influence hepatic outcomes. The bacterial species responsible for amino acid metabolism in our study have previously been associated with altered BCAA metabolism in a study in obese youths with or without MASLD [62] highlighting that the presence and function of these species are relevant for future research and microbe-targeted therapies in MASLD.

In our study, butyrate production pathways were enriched in healthy controls compared to participants with MASLD. Butyrate is a SCFA that has beneficial effects on intestinal barrier health, inflammatory and immune processes [63]. Associations have been reported between decreased butyrate producing bacteria [64] and altered inflammatory markers [65] in individuals with MASLD. As butyrate is a by-product of dietary fibre fermentation, we propose that butyrate production was enhanced in healthy controls due to higher dietary fibre intake. However, considering that butyrate also increases energy harvest by gut microbes [66], its role in metabolic syndrome and associated conditions such as MASLD is complicated and requires further elucidation.

Most consistently, dietary fats and sugars were associated with bacterial species abundance and functional pathway expression. Dietary fats and sugars were previously not considered to substantially influence colonic microbiota composition due to their absorption being primarily in the small intestine. It is now recognised that a dietary load of nutrients such as fats and sugars beyond what is able to be absorbed in the small intestine, may reach the large intestine and act as substrate for colonic microbes [67]. There is also potential for nutrient degradation earlier in the gastrointestinal tract to produce metabolites or secondary byproducts that subsequently influence the characteristics of colonic microbes. Together with evidence that small intestinal bacteria can differ in individuals with and without chronic liver disease [68], this suggests that interactions between gut microbes and diet in MASLD need to be considered and investigated across the entire gastrointestinal tract.

The current research aligns with recent practice guidelines for MASLD which encourage dietary intervention including caloric deficit in patients with overweight or obesity, and adoption of Mediterranean dietary patterns including limited carbohydrates (especially sugars), high dietary fibre and replacing saturated with unsaturated fat [8, 40−69]. The current research offers new hypotheses regarding dietary management of MASLD, by providing evidence that current recommendations may exert benefits, at least in part, via gut microbiota modulation. Key findings and comparisons with current guidelines and implications for dietetic practice are summarised in Table 5.

Whilst systematic review and meta-analysis have supported the role of Mediterranean diet in reducing hepatic steatosis and indices of MASLD severity [10–41], it remains unclear whether these relate to known cardiovascular and metabolic health benefits or if hepatic-specific alterations also exist in MASLD. The Mediterranean diet is accepted as anti-inflammatory and effective for reducing systemic inflammation [23]. The gut-liver axis is one pathway for potential hepatic specific influences, with the gut microbiota facilitating known pro or anti-inflammatory effects of specific foods compounds through metabolite production and altered intestinal permeability [28]. Our study found that Mediterranean diet dietary constituents including VLCN3 and dietary fibre were consumed less and associated with less abundant bacterial species in the MASLD group. Furthermore, these nutrients were negatively associated with inflammatory markers hs-CRP, TNF-α and CK-18, suggesting an interaction between diet, microbes and systemic inflammation. Endotoxemia and associated systemic inflammation characteristic of MASLD [70], is exacerbated by high dietary sugar and fat intake [23] and potentially reversible by replacing saturated to unsaturated fat intake [23]. It is likely multiple mechanisms are at play, with VLCN3 and polyphenols likely reducing systemic inflammation, which may be further offset by dietary fibre modulation of the microbiota. Further research is needed to elucidate exact mechanisms between constituents of the Mediterranean diet, the microbiota and endotoxemia and inflammation relevant to MASLD.

This study analyses had both strengths and limitations. This is one of the first studies to triangulate microbiota, dietary data, and clinical data in people with MASLD compared to healthy controls, using dietary data proximal to microbiota sampling. However, due to the sample size, sub-group analysis was not conducted for those with differing disease severity or co-morbidities. There were also significant differences between groups at baseline which may have influenced results, however these differences were accounted for in all statistical analyses, with no associations evident between BMI, prescription medication use or ethnicity and bacterial relative abundance or functional pathways. These limitations highlight that future studies with larger, more diverse sample sizes are required to ensure generalisability of results. MASLD studies may be confounded by the coexistence of other metabolic conditions, which were not differentiated in this study. The change in nomenclature from NAFLD to MASLD during the timeline of this study meant to participants did not meet the criteria for MASLD. We acknowledge that by including these participants in the analysis may mean that study findings are not specifically applicable to MASLD as per the new definition. Future studies with larger sample sizes are needed to confirm the specificity of findings and more accurately attribute effects. Limitations of dietary assessment in microbiota research also limit this research. The Australian food composition databases do not currently allow for assessment for amino acid intake, phytonutrients or specific dietary fibre types, and therefore could not be assessed. Expansion of nutrient databases to assess all dietary components relevant to the gastrointestinal tract is essential for specific and valid assessment of these interactions.

## Conclusions

In conclusion, this study reinforces that the gut microbiome differs in individuals with MASLD compared to healthy controls. The current study is the first to investigate associations between the gut-liver axis and diet and suggest dietary constituents that may influence gut microbe characteristics and subsequent hepatic health outcomes. A key finding was the inverse association between dietary constituents aligned with Mediterranean dietary principles and gut microbes and functional pathways proposed to contribute to MASLD. The Mediterranean diet is known to be accepted in individuals with MASLD [10] and is likely to benefit comorbidities [10]. This reinforces the promotion of a Mediterranean diet for individuals with MASLD, with a focus on the quality of dietary fats and carbohydrates and their food sources. Metabolomics work is required to better understand the hepatic implications of the gut microbiota differences evident in this work. Future research focused on microbe relevant nutrients will elucidate mechanisms of diet related influences on the gut-liver axis in order to optimise nutrition therapy for the amelioration of MASLD.

## Electronic supplementary material

Below is the link to the electronic supplementary material.

Supplementary Table 1: Excel spreadsheet (.xls) *Associations between diet*, *clinical characteristics and inflammatory markers*. This spreadsheet provides correlation analysis outcomes between diet, clinical characteristics and inflammatory markers in individuals with MASLD and healthy controls

Supplementary Table 2: Excel spreadsheet (.xls) *Associations between different taxonomic composition and MASLD status*. This spreadsheet provides correlation analysis outcomes between relative abundance of microbial species and MASLD status (disease vs. control).

Supplementary Table 3: Excel spreadsheet (xls) *Associations between microbiome functional pathways*, *MASLD status and dietary intake.* This spreadsheet provides correlation analysis between microbial functional pathways and dietary variables in individuals with MASLD and healthy controls.

## Data Availability

The dataset supporting the conclusions of this article is available in the NCBI BioProject repository, accession number PRJNA1188624. The NCBI SRA record and phenotype information is accessible at the following link: https://www.ncbi.nlm.nih.gov/sra/PRJNA1188624.

## Abbreviations

MASLD: Metabolic-associated steatotic liver disease
MAS: Metabolic dysfunction-associated steatohepatitis
BMI: Body mass index
MaAsLin: multivariate associations with linear models
PCoA: Principal coordinates analysis
Fib-4: Fibrosis-4 score
MFS: Metabolic dysfunction associated steatotic liver disease Fibrosis Score
ALT: Alanine aminotransferase
AST: Aspartate aminotransferase
IL-1β: Interleukin 1β
TNF-α: Tumour Necrosis Factor-a
CK-18: Cytokeratin-18
hs-CRP: High sensitivity C-reactive protein (hs-CRP)
IL-6: Interleukin-6
SCFA: Short chain fatty acids
BCAA: Branched chain amino acids
PUFA: Polyunsaturated fats
EPA: Eicosapentaenoic acid
DHA: Docosahexaenoic acid
DPA: Docosapentaenoic acid
ALA: Alpha-linolenic acid
